# Understanding the Dynamics of Domestic Violence During the First Year of the Pandemic: An Integrative Review

**DOI:** 10.1177/15248380241277788

**Published:** 2024-09-24

**Authors:** Ana Cunha, Mariana Gonçalves, Marlene Matos

**Affiliations:** 1 Center of Investigation in Psychology, School of Psychology, University of Minho, Braga, Portugal

**Keywords:** integrative review, PRISMA, COVID-19, domestic violence, prevalence, risk factors, help-provision

## Abstract

This integrative review aims to analyze and synthesize existing literature to inform our understanding of the multifaceted dimensions of domestic violence during the first year of the COVID-19 pandemic, using a holistic and ecological framework. Utilizing the Preferred Reporting Items for Systematic Reviews and Meta-Analyses (PRISMA) study design, searches were conducted on six databases, yielding a final sample of 58 articles. The study comprehensively overviews North America, South America, Asia, Europe, Africa, and worldwide research. The literature reveals an alarming increase in domestic violence victimization during the pandemic in most regions and studies, exacerbating pre-existing vulnerabilities. The increase in domestic violence during the pandemic is linked to ecological factors such as lower physical and mental health, rising substance use, and financial stress, which heightened individuals’ vulnerability. Lockdowns exacerbated these issues by increasing confinement in homes, disrupting support services, and limiting victims’ access to help. Barriers to help-seeking and amplified personal and professional stressors at the care level are identified. Advocacy for improved awareness, cooperation, and inclusive national and institutional policies emerges. This study underscores the urgency of empirical research to generate reliable data on the pandemic’s impact on domestic violence. The findings of this study highlight the importance of understanding unique factors affecting specific groups, as well as informing prevention efforts and targeted interventions. Recognizing the mutual benefit of research–practice partnerships is crucial in addressing and preventing domestic violence. This research contributes to a deeper understanding of domestic violence during the pandemic’s first year, guiding empirically informed interventions and policy changes.

## Introduction

Domestic violence, perpetrated through abuse of power and with serious physical and mental consequences (Machado et al., 2016), violates the fundamental human rights and dignity of the victim as well as of the entire community. Although it may be committed against any member of the family (e.g., elderly people), most of the current international research has focused on intimate partner violence (Hawcroft et al., 2019).

Domestic violence is highly prevalent worldwide (e.g., 1.9%–70% prevalence toward women; Alhabib et al., 2010), and increasing rates were of particular concern during the global COVID-19 outbreak. The pandemic prompted many countries to enact nationwide lockdowns as a preventive strategy against the virus’s transmission. Nonetheless, the repercussions of these widespread lockdown measures proved to be substantial and widespread. These consequences included economic upheaval, mental health difficulties, interruptions in education, pressures on healthcare systems, increased social isolation, and a global upsurge in domestic violence cases.

The necessity of staying at home as a protective measure against the virus inadvertently heightened the vulnerability of individuals to violence, with factors like isolation playing a key role in both the frequency and severity of such abuse (World Health Organization [WHO], 2020). While being required to stay confined with their abusers, and away from those who can provide support (e.g., family support network, neighbors, and support institutions), the home became a very dangerous place for victims of domestic violence living under these circumstances (Marques et al., 2020; Mazza et al., 2020; M’Jid, 2020; Platt et al., 2020; Viero et al., 2021). China, for instance, reported a three-fold increase in violence, while France witnessed a 30% rise. Similarly, Brazil estimated a 40% to 50% jump in domestic violence cases, and Spain observed a trend of intimate partner violence-related homicides (Campbell, 2020). Furthermore, a comparison between April 2020 and the same timeframe in the previous year revealed a 60% surge in emergency calls from women who experienced violence from their intimate partners in the European member states of the World Health Organization (WHO) (Mahase, 2020). However, the findings on the prevalence of domestic violence during the pandemic present a nuanced and mixed landscape. While some studies report an alarming surge in domestic violence cases, attributing the increase to the heightened stressors, economic uncertainties, and confined living conditions brought about by the pandemic, others suggest more equivocal results (Piquero et al., 2021). This nuanced picture underscores the importance of considering diverse perspectives and methodological approaches when comprehensively understanding the impact of the pandemic on domestic violence prevalence.

To understand the link between the COVID-19 pandemic and domestic violence, we based this review on the WHO ecological framework (Krug et al., 2002). This framework can clarify how the changes in domestic violence incidence during the pandemic were influenced by the complex interaction of individual, relationship, and environmental factors, all of which were significantly impacted by the pandemic. This holistic approach highlights that while the lockdown measures and social isolation played a key role in both the frequency and severity of such abuse (WHO, 2020), other consequences of the pandemic, such as economic stressors and disrupted social services, also contributed to the changes in the domestic violence phenomenon.

Previous findings have indicated an increase in domestic violence rates after natural disasters or public health emergencies (Onyango et al., 2019; Schumacher et al., 2010). Furthermore, while the expected increase in domestic violence may not be visible immediately, the risks associated are very high and will likely remain that way in the coming months (Campbell, 2020). Current recommendations call for continuing, concerted efforts against this phenomenon, especially during emergencies like the COVID-19 pandemic, something also highlighted and reinforced in the European Union Strategy on Victims’ Rights 2020 to 2025 (European Commission, 2020).

The confinement measures, economic uncertainties, and heightened stressors brought about by the pandemic have raised questions about the prevalence, risk factors, and impact of domestic violence within the confines of home. To comprehensively address these critical inquiries, this systematic review aims to synthesize and analyze existing literature to inform our understanding of the multifaceted dimensions of domestic violence during the first year of the pandemic. We undertook this integrative review with the following objectives: (a) Summarize the prevalence and reporting of domestic violence: This review provides a comprehensive overview of the prevalence and reporting patterns of domestic violence during the initial year of the COVID-19 pandemic. (b) Summarize risk factors and major concerns: This review systematically examines the literature to identify risk factors associated with the occurrence and escalation of domestic violence during the pandemic, shedding light on the complex interplay of individual, relationship, and social/structural determinants. (c) Summarize major concerns on help-seeking and help-provision: To examine how the pandemic has influenced help-seeking and help-provision, considering the accessibility and adequacy of support services during this challenging period. (d) Synthesize evidence to inform practice, policy, and future research: Finally, this systematic review synthesizes the available evidence to provide actionable insights for practitioners, policymakers, and researchers. By critically evaluating the existing literature, we aim to inform the development of evidence-based practices, shape policy decisions, and identify gaps in the current understanding of domestic violence during the pandemic for future research endeavors.

Each objective contributes a piece to the overall understanding of the issue, creating a comprehensive narrative that can guide not only researchers but also practitioners, policymakers, and advocates in addressing the complexities of domestic violence during the unique circumstances of the pandemic’s first year. While a more focused review might be appropriate for specific questions, an inclusive and ecological approach is beneficial when dealing with a complex and socially significant topic like domestic violence during a global crisis.

## Method

### Study Design

Authors based their research on the Preferred Reporting Items for Systematic Reviews and Meta-Analyses (PRISMA; Moher et al., 2009). We conducted searches on six databases: Science Direct, Medline/PubMed, Scopus, PsycArticles/PsycINFO, and Web of Science. Articles selected corresponded with a paired combination of the following two keyword categories: (“domestic violence” OR “intimate partner violence” OR “interpersonal violence” OR “violence against women” OR “gender-based violence” OR “battered women” OR “violence against men” OR “family violence” OR “domestic abuse”) AND (COVID-19 OR coronavirus OR pandemic OR SARS-CoV-2 OR quarantine OR lockdown).

### Inclusion Criteria

This systematic review considered articles investigating violence within domestic settings in adults (including women and men), during the COVID-19 outbreak. The search parameters for this review were time-bound, covering the period from January 2020 to March 2021, to ensure a comprehensive retrieval of relevant literature. Inclusion criteria comprised empirical studies, specifically excluding commentaries, letters to editors, and theoretical articles. The focus was solely on peer-reviewed journal publications, with exclusion criteria encompassing dissertations, books/chapters, and governmental reports. No limitations were imposed based on the research design. In the case of review articles, inclusion required the presence of one or more outcomes related to domestic violence, even if the primary focus extended beyond this subject. Eligible studies included those involving victims of domestic violence, police or other reports, the general population (e.g., prevalence studies), or alternative sources of data collection. The review did not set restrictions on sample size, study objectives, or statistical methodologies, aiming for a comprehensive overview of the research landscape. Geographical setting or publication status were not restricted, with the only language requirement being that studies must be available in English, Portuguese, French, or Spanish.

### Selection of Studies

**Figure 1.**
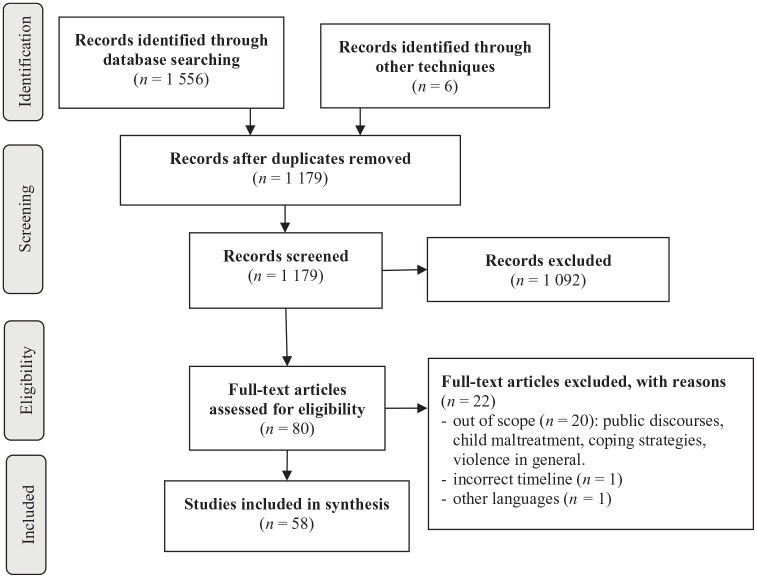
Process flow diagram.

These initial searches produced 80 articles from PsycINFO/PsycArticles, 199 articles from Web of Science, 241 articles from Medline/PubMed, 327 articles from Sage, 340 articles from Scopus, 369 articles from ScienceDirect, and 6 articles using other techniques (e.g., hand search). These results were combined into a single list using a Rayyan software (Ouzzani et al., 2016), and duplicates were removed, leaving 1,179 articles. In the first stage, two authors screened first titles and abstracts to obtain articles that could potentially meet inclusion criteria that were later accessed in full text for further assessment (AC and MG). An additional manual search was conducted from the reference lists of each included study. The full text of 80 articles was then independently analyzed and evaluated for inclusion. Following this review process, we arrived at a final sample of 58 articles (see Figure 1 for a visual representation of our search process).

### Data Extraction and Analysis

Two researchers (AC and MG) independently extracted data and compared findings to ensure consistency, using a charting form to organize key variables: article title, authors, year of publication, the country where the survey was conducted, purpose/aim related to domestic violence, study design, population, screening methods used, and key findings, including recommendations for practice, policy, and research. The term “domestic violence” was employed as a broad descriptor for violence occurring within domestic contexts (e.g., elder abuse, intimate partner violence, violence against women). Data from North America, South America, Asia, Europe, Africa, and worldwide research were retrieved. The results provided below are organized into these domains for clarity of reporting. Article summaries are listed in Table 1.

**Table 1.** Data Chart Original Research.

Article and Country (in Alphabetical Order)	Themes	Study Design and Population	Key Findings	Recommendations
Abdel Rahman (2021) Egypt	Incidence of DV; risk factors and major concerns	Quantitative design Community-based, 312 married people (167 men and 144 women)	High levels of DV. Risk factors: years of marriage and gender.	Designing and researching counseling and psychotherapy programs, both in traditional settings and online platforms.
Abuhammad (2021) Jordan	Incidence of DV; risk factors and major concerns	Quantitative design Community-based, 687 pregnant women	40% of DV, with 9.8% experiencing previous violence. Risk factors: Unemployment and marriage.	Create educational prevention programs for health. Enhance communication strategies. Utilize diverse research methodologies, such as qualitative or mixed methods designs.
Abujilban et al. (2022) Jordan	Incidence of DV; risk factors and major concerns	Quantitative design Community-based, 215 pregnant women	Higher incidences of violence before the quarantine. Risk factors: marital conflict, verbal fighting, and not understanding each other.	Supervise and educate women with a history of violence. Introduce evidence-based interventions and support services. Initiate nationwide prevention initiatives, targeting all social classes through random sampling and engagement with care providers.
Agüero (2021) Peru	Incidence of DV	Quantitative design Reports from a national helpline	The incidence rate of the calls increased by 48% between April and July 2020, with effects increasing over time.	Identify policies to mitigate unintended effects of stay-at-home orders. Examine the impact of financial aid on reducing DV during the pandemic.
Akel et al. (2022) Lebanon	Incidence of DV; risk factors and major concerns	Quantitative design Community-based, 86 married couples	41.9% physical, 40.7% psychological, and 38.4% sexual abuse. Higher levels of anxiety, stress, depression, and low self-esteem.	Increase the public’s awareness. Work on solutions to decrease the risk and improve support. Empowerment of (non)governmental organizations.
Aolymat (2021) Jordan	Incidence of DV	Quantitative design Community-based, 200 women	20.5% of women suffered from increased domestic abuse during the COVID-19 pandemic.	To introduce domestic support and reproductive health services and measures during pandemic situations.
Arenas-Arroyo et al. (2021) Spain	Incidence of DV; risk factors and major concerns	Quantitative design Community-based, 13,786 women	23% increase in DV during the lockdown. Risk factors: at least one of the members of the couple is locked and the economic stress.	Special attention should be devoted to couples without previous levels of violence, with children and of low socioeconomic status.
Campos et al. (2020) Brazil	Help-seeking and help-provision	Qualitative design Service providers, 3 women	The social vulnerability of women intensified. Implementation of new emergency measures and care protocols in protective services. Increase deprivation of remote communication.	Expanding the intersectoral networks and mental health services.
Cannon et al. (2021) USA	Incidence of DV; risk factors’ and major concerns	Quantitative design Community-based, 374 men and women; race/ethnicity included	10.4% experienced DV. Risk factors: COVID-19 income loss, renter status, nutritional stress. DV group reported higher stress and a greater need for help.	Boost DV awareness via social media. Partner COVID-19 testing sites with DV organizations. Strengthen communication tools, involve agencies in policymaking, explore online DV interventions’ protective role, and adopt an intersectional approach.
E. S. Chang and Levy (2021) USA	Incidence of DV; risk factors and major concerns	Quantitative design Community-based, 897 older women and men; racial/ethnic minorities included	21.3% elder abuse victimization since the beginning of the pandemic. Risk factors: younger, large households, poorer health, lower sense of community, lower adherence to physical distance, higher levels of financial strain.	Preparedness and training for medical professionals. Develop community support mechanisms. Collaborative efforts and awareness. Emphasize collaborative efforts to enhance victims’ help-seeking knowledge and behavior. Telemedicine utilization.
Y. R. Chang et al. (2020) Korea	Incidence of DV	Quantitative design Reports from a trauma center	The prevalence of domestic incidents increased significantly in March 2020 (31% vs 5-year average of 16.1% and every month of March 18%).	The development of social consensus, policies, and psychological support services. Study the psychological impact of the COVID-19 pandemic and social distancing measures.
de la Miyar et al. (2021) Mexico	Incidence of DV	Quantitative design Reports from a court	A decrease of 4.9 DV reports per 100,000 inhabitants (77% lower than the control period).	Not available.
Di Franco et al. (2021) Italy	Incidence of DV; risk factors and major concerns	Quantitative design Reports from an ER	Diagnosis of DV was 0.6% among emergency admissions. Highest percentages of abortions, daily activities impairment, psychosomatic symptoms, and anxious-depressive symptoms.	To implement screening protocols: traumatic injuries, psychosomatic symptoms, and anxious-depressive symptoms. Recognize the power of detection of DV by the WHO questionnaire.
Evans et al. (2021) USA	Incidence of DV	Quantitative design Service reports from police data	11% increased occurrence of DV after shelter-in-place orders.	Tackle unemployment and financial hardship. Allocate resources to DV prevention and intervention. Adopt a public health approach. Investigate DV injuries in healthcare and support services demand. Analyze considering pandemic-related movement restrictions.
Every-Palmer et al. (2020) New Zealand	Incidence of DV	Quantitative design Community-based, 941 men, 1,063 women, 6 gender diverse; gender minorities included	9.1% of participants had directly experienced some form of family harm over the lockdown period. 3.9% reported witnessing family harm in which they were not the victim.	Targeted communication for priority groups. Strengthen mental health with free e-therapies. Collect robust mental health data. Research priority groups using qualitative methods. Examine vulnerabilities and protective factors.
Fawole et al. (2021) Nigeria	Incidence of DV; risk factors and major concerns; help-seeking and help-provision	Qualitative design Reports from help services and media reports	DV was occurring before the lockdown but increased in severity or involved new types of violence. Stressors identified were custody of children, disrupted income generation, and economic stressors. The lockdown disrupted women’s support.	Aid women in emergencies for healthcare and legal support. Encourage family and community support for abused women in crises. Conduct surveys or qualitative interviews. Enlist larger, representative samples.
García-Fernández et al. (2021) Spain	Risk factors and major concerns	Quantitative design Community-based, 520 men and 1,115 women	Women who had experienced DV had significantly higher levels of depressive symptoms than those who had not.	Address the gender impact of outbreaks, as well as the benefit of paying special attention to women who suffer DV to ensure their emotional well-being.
Gebrewahd et al. (2020) Ethiopia	Incidence of DV; risk factors and major concerns	Quantitative design Community-based, 682 women	24.6% prevalence of DV against women. Risk factors: uneducated housewives, aged younger than 30 years, women with arranged marriages, and women with husbands aged “between” 31 and 40.	Identify high-risk individuals to strengthen the link between social and national health systems, family laws as well as police investigations to prevent the high impact of VD against women.
Gerell et al. (2020) Sweden	Incidence of DV	Quantitative design Reports from police data	Indoor violence, including DV, dropped during weeks 9–11 (March 11,, 2020) and since appears to have stabilized at a level that is slightly lower than expected.	Formal comparisons between countries.
Ghimire et al. (2020) Nepal	Incidence of DV; risk factors and major concerns	Quantitative design Community-based, 283 men and 271 women	8% reported DV victimization. Risk factors: younger age, unmarried but living with a spouse, lower education, and prior mental disorder. Poor mental well-being, increased tobacco/alcohol use during lockdown linked to victimization.	Highlight vulnerable groups and manage interpersonal violence effectively. Identify victims by gender, age, living arrangement, and education to address the issue. Use a large sample, targeting vulnerable groups like children, adolescents, and women. Implement longitudinal studies.
Gosangi et al. (2021) USA	Incidence of DV	Quantitative: cross-sectional Reports from an ER	The overall number of patients who reported DV decreased during the pandemic, the incidence of physical DV was 1.8 times greater, and the the incidence of high-risk abuse defined by mechanism was two times greater in 2020.	Radiologists and other healthcare providers should proactively participate in identifying victims of DV and reaching out to vulnerable communities as an essential service during the pandemic and other crises.
Hamadani et al. (2020) Bangladesh	Incidence of DV	Quantitative design Community-based sample: 2,414 mothers	90% of DV during the lockdown. Increased emotional (66%–68.7%), physical (56%), and sexual (50.8%) violence since the lockdown.	Provide welfare for family income support. Strengthen local community services and support health workers. Introduce modules on factors like women’s empowerment. Use qualitative research to understand experiences during lockdown.
Haq et al. (2020) Pakistan	Incidence of DV; risk factors and major concerns	Quantitative design Community-based sample: 389 women	35% revealed to suffer from violence. 17% reported having faced emotional violence up to 12 times. Risk factors: extended household, higher number and taking care of children, poor relationship with husband, unemployment, anxiety, and not feeling empowered.	Implement outreach policies for victims’ health, safety, and empowerment. Achieve prevention through effective policing, strict legislation, and education. Conduct similar investigations in countries reinforcing stereotypical women’s roles and lacking gender equity.
Holland et al. (2021) USA	Incidence of DV	Quantitative design Reports from an ER	DV was higher in mid-March through October 2020, during the COVID-19 pandemic, compared with the same period in 2019.	Implement evidence-based interventions. Introduce virtual services. Expand societal and community-level prevention efforts. Include additional analyses to assess observed changes over time and during longer periods. Incorporate additional data.
Hsu and Henke (2022) USA	Incidence of DV	Quantitative design Reports from police data	Staying at home due to COVID-19 increased DV by over 5% on average from March 13 to May 24, 2020.	A need to give victims resources and a place away from their abusers such as a shelter.
Jetelina et al. (2021) USA	Incidence of DV; risk factors and major concerns	Quantitative design Community-based, 1,196 women, 592 men (missing data *n* = 246); race/ethnicity included	18% screened positive for DV, with 17% stating that victimization worsened. Sexual (2.31 times higher) and physical violence (4.38 times higher). Risk factors: job/income change due to the pandemic.	Prioritize victim services, secure funding, implement DV screening in health facilities, train providers in trauma-informed care, and raise community awareness. Conduct multisectoral research, gather nationally representative quantitative data, and utilize qualitative insights for improved public health campaigns and service accessibility.
Jung et al. (2020) Germany	Incidence of DV	Quantitative design Community-based, 2,946 women, 539 men, 60 diverse or missing	5% of all participants reported experiencing DV. More verbal and physical violence was reported by women and men; women but none of the men reported more sexual violence.	To continuously monitor the mental health of the public during this pandemic and its aftermath to identify associated protective factors. Control for social desirability effects.
Koshan et al. (2020) Canada	Help-seeking and help-provision	Qualitative design Reports from courts	Limited awareness of increased risks for survivors during COVID-19. Emphasis on incident-based physical violence rather than pre-pandemic coercive control patterns. Challenges in proving DV and obtaining court orders due to procedural complexities and reduced service availability.	Reforms are needed not only to address the pandemic and the increase in DV that is anticipated to exist well beyond the life of COVID-19, or to respond more effectively to future viral pandemics but also to ensure access to justice for women and children. The need to deepen the education of judges and lawyers about DV.
Krishnamurti et al. (2021) USA	Incidence of DV	Quantitative design Community-based, 552 women before the shelter-in-place; 407 during the shelter-in-place; race/ethnicity included	Similar levels of physical violence, sexual violence, and psychological abuse before and during the shelter-in-place order.	Care providers should consider the unique needs of pregnant women, to provide them with opportunities for mitigating pregnancy-related risks. Technologies may offer patients an additional method for communicating.
Leslie and Wilson (2020) USA	Incidence of DV; risk factors and major concerns	Quantitative design Reports from police data	DV service calls rose by 7.5% in the 12 weeks post-social distancing, peaking at a 10% increase in the first 5 weeks, particularly affecting households without a prior DV history.	Not available.
Lyons and Brewer (2022) Worldwide	Incidence of DV; risk factors and major concerns; help-seeking and help-provision	Qualitative design Discussion forum posts written by victims	DV victim experiences during lockdown and the pandemic included abusers using COVID-19 threats, service disruptions, delayed attempts to leave, and increased abuse/distress due to factors like financial stress and isolation.	Support victims in recognizing and responding to diverse DV. Inform victims about social distancing exceptions. Conduct national campaigns for safety information. Increase law enforcement awareness of DV risks. Future studies: consider minority groups, same-sex relationships, and men with female perpetrators.
Mahmood et al. (2022) Iraq	Incidence of DV	Quantitative design Community-based, 346 married women	Violence increased during lockdown: any violence (32.1%–38.7%), emotional abuse (29.5%–35.0%), physical violence (12.7%–17.6%), forced sexual intercourse (6.6%–9.5%).	Boost awareness and collaboration for social stability. Future research: focus on vulnerable women, including those with lower education and socioeconomic status, and displaced/refugee populations.
Maji et al. (2022) India	Incidence of DV	Qualitative design Newspaper and media/digital data	A major increase in DV cases was observed during the COVID-19 period as compared to the previous years.	More strict and effective legal provisions. To educate and prepare a gender-egalitarian society. More cooperation between governments, law enforcement agencies, non-governmental organizations, and the public. More studies and effective intervention programs.
McLay (2022) USA	Incidence of DV	Quantitative design Reports from police data	March 2020: Fewer cases (51.26% vs. 48.74%). Arrested cases were 20% less likely, residential cases 22% more likely, and child victims 67% less likely.	Protect children in residential areas. Fund victim relocation services. Enhance data for analysis. Compare pandemic DV reports to usual rates. Encourage open data sharing.
Mohler et al. (2020) USA	Incidence of DV	Quantitative design Reports from police data	Significant increases in DV calls.	Compare calls-for-service and crime numbers with local data on new infections. Address study limitations as additional data become available (e.g., generalization).
Morgan and Boxall (2020) Australia	Incidence of DV; risk factors and major concerns	Quantitative design Community-based, 7,514 women in a current cohabiting relationship (6,925 no prior violence, 521 prior violence)	2.9% reported first-time partner violence. 67% with prior violence experienced repeat incidents. Risk factors: reduced social interaction, less time at home, financial stress.	Not available.
Muldoon et al. (2021) Canada	Incidence of DV	Quantitative design Reports from an ER sexual assault and domestic violence program	ER admissions for sexual assault and DV decreased during COVID-19: 53.49% decrease in sexual assault and 48.45% in physical assault. Significant increases in psychological abuse (11.69%–28.57%) and assaults outdoors (5.19%–22.86%).	Require multi-sectoral collaborations. Support seeking urgent or alternative care models. Employ difference-in-differences study designs. Gather evidence to provide adequate support for individuals experiencing violence.
Naghizadeh et al. (2021) Iran	Incidence of DV; risk factors and major concerns	Quantitative design Community-based, 250 pregnant women	During COVID-19, 35.2% experienced DV: emotional (32.8%), sexual (12.4%), and physical (4.8%). Risk factors: self-rated health, reduced spouse income, aggressive spouse behavior, and disease impact on the relationship. Lower mental health and quality of life for those exposed to violence.	The health sector must implement measures to reduce violence risk. Governments should develop plans addressing violence against women during COVID-19, allocating necessary resources, and adopting strategies for implementation.
Nix and Richards (2021) USA	Incidence of DV	Quantitative design Reports from police data	DV-related calls for service generally increased the week stay-at-home orders went into effect and declined throughout the remainder of the year.	Train police in DV incidents and trauma-informed interviewing. Direct law enforcement to mental health services. Promote a culture of inter-agency data sharing. Explore neighborhood-level changes in DV. Examine trends in DV help-seeking, including law enforcement and hotline calls.
Nnawulezi and Hacskaylo (2022) USA	Help-seeking and help-provision	Qualitative design 840 service providers	Practitioners focused on managing housing programs, addressing resource loss for survivors, ensuring staff safety, and maintaining organizational operations during COVID-19.	Ensure low-barrier shelter access with personalized services. Offer flexible financial aid and support mutual aid for housing and economic stability. Provide survivor-led safety planning with choices. Establish safe housing options in various models and settings. Cultivate a trauma-informed organizational culture promoting autonomy.
Olding et al. (2021) UK	Incidence of DV	Quantitative design Reports from an ER	DV-related injuries increased during the lockdown period, but the proportion of DV among total penetrating trauma decreased, remaining the most common cause overall.	Identify and assist those most at risk. Research the impact of social restrictions on undiagnosed and diagnosed mental health conditions.
Pal et al. (2021) India	Incidence of DV; risk factors and major concerns	Quantitative design Community-based, 138 men and 133 women; sexual orientation included	DV reports significantly increased during the lockdown, particularly among those who lost their jobs due to the restrictions.	Development of culture-sensitive interventions.
Payne et al. (2020) Australia	Incidence of DV	Quantitative design Reports from governmental data	The DV rates remained unchanged.	Continued data collection and analyses. Track any changes in crime rates when these restrictions are loosened and potentially eliminated. Combination of quantitative and qualitative data.
Piquero et al. (2020) USA	Incidence of DV	Quantitative design Reports from police data	An increase in DV in the first 2 weeks after the stay-at-home order was implemented but then a decrease thereafter.	Not available.
Raj et al. (2020) USA	Risk factors and major concerns	Quantitative design Community-based, 1,139 women and 942 men; race/ethnicity and sexual orientation included	DV was associated with greater odds of mild and moderate mental health symptoms; sexual violence was associated with increased odds of mild and severe mental health symptoms.	Offer financial aid and job opportunities. Ensure mental health resources. Address socially vulnerable populations. Strengthen virtual social support. Collect regular longitudinal data for accurate mental health reporting.
Rhodes et al. (2020) USA	Incidence of DV	Quantitative design Reports from an ER; race/ethnicity included	Increase in assaults was found during the COVID-19 lockdown, particularly during the period after school closures.	Improve support programs. Maintaining and prioritizing shelters, hotlines, websites, and other support services. Improve public awareness. Follow-up studies will use expanded time frames and larger patient numbers.
Ribeiro-Junior et al. (2021) Brazil	Incidence of DV	Quantitative design Reports from police and health data	DV decreased during the first half of 2020, with a further reduction in this rate with the increase in COVID-19 cases.	More analysis as the pandemic evolves.
Sabri et al. (2020) USA	Incidence of DV; risk factors and major concerns; help-seeking and help-provision	Qualitative design 17 service providers Victims-based, 45 immigrant women	Lockdown increased DV severity and frequency. Risk factors: partners at home, increased control. The lockdown impacted survivors’ mental health and finances. Altered service demand and effectiveness.	Tailored interventions, standardized healthcare IPV screening, culturally trained staff, funded services, diverse interactions, community ties, DV service access, assess strategy, study the pandemic impact on immigrant survivor care, implement culturally tailored approaches.
Sediri et al. (2020) Tunisia	Incidence of DV; risk factors and major concerns	Quantitative design Community-based, 751 women	Lockdown increased VD from 4.4% to 14.8%. Pre-pandemic violence was linked to higher lockdown violence (73% vs. 12% without DV history). VD was linked to higher depression, anxiety, and stress.	Enhance psychological intervention, urgent crisis protection strategies for women, family education, and civil society-government collaboration for lasting change.
Sharma and Khokhar (2022) India	Incidence of DV	Quantitative design Community-based, 55 women and 39 men	7.4% faced DV during lockdown. Of these, 85.7% reported an increase in DV during lockdown.	Improve health-care awareness about increased DV during lockdown. Screening in health-care context. Disseminate helpline numbers/strategies to patients. Improve community awareness.
Singh (2020) India	Incidence of DV; risk factors and major concerns	Quantitative design Community-based, 165 women	Women experienced high levels of DV. Risk factors/predictors: financial crisis, extramarital affairs, and a dominant nature.	Raise awareness. Incorporate social sensitivity and consciousness-raising activities related to DV in schools.
Speed et al. (2020) UK	Help-seeking and help-provision	Qualitative design 51 service providers	Capacity boosted, limited justice for at-risk victims due to reduced services, and inadequate funding; only 17 of 40 organizations provided support during the outbreak; concerns about accessing justice, trial delays, and communication issues with victims.	Funding and technical support across sectors. Public campaigns about resources.
Stephenson et al. (2022) USA	Incidence of DV; risk factors and major concerns	Quantitative design Community-based sample: 696 men; race/ethnicity and sexual orientation included	12.6% of participants reported experiencing DV, with 5.3% reporting that this was their first experience. Risk factors: substance use or alcohol intake, skipping meals more frequently, and reported higher levels of anxiety.	Increased need for DV resources for GBMSM. More awareness among services for specific groups of victims Understand the forms, functions, and content of DV resources that can be provided to GBMSM.
Tadesse et al. (2022) Ethiopia	Incidence of DV; risk factors and major concerns	Quantitative design Community-based, 589 married women; race/ethnicity included	22.4% overall prevalence of at least one form of DV. Risk factors: being illiterate, having illiterate husband, having substance user, and community tolerant attitude to violence.	Enhancing gender equality. Use community- and institution-based approaches to prevent DV. Accessibility of education for women. Qualitative studies to explore the socio-cultural practices that support women violence in the community.
Teshome et al. (2021) Ethiopia	Incidence of DV; risk factors and major concerns	Quantitative design Community-based, 464 pregnant women	9.5% reported DV exposure within the year; 20.4% reported increased DV post-pregnancy; 18.2% perceived increased DV after COVID-19; risk factors include drug and alcohol use.	Not available.
Tierolf et al. (2021) Netherlands	Incidence of DV; help-seeking and help-provision	Mixed design Victims-based, 159 pre-lockdown (105 women and 54 men), 87 post-lockdown (54 women and 33) 13 service providers	Violence frequency remained unchanged pre and post-lockdown, but serious violence persisted. The pandemic altered family life, causing stress. Support shifted to virtual formats (e.g., video and phone calls).	Better prepare and train staff in forms of digital contact. Ensure that the technical conditions are in place to enable video calling for professionals. Guidelines and information about digital contact (e.g., about privacy or the extent to which assistance could be provided).
Wood et al. (2022a) USA	Incidence of DV; risk factors and major concerns; help-seeking and help-provision	Mixed design Victims-based, 42 women and 8 men; race/ethnicity included	Safety concerns related to violence, stalking, threats or past abuse. Concerns related to service and support needs (i.e., service access, virtual services). Broader economic and health concerns (i.e., health and health care access, economic challenges like unemployment, problems obtaining food and other supplies, lack of childcare and school).	Increasing access and availability of services. Staff training is needed on diverse platforms to meet survivor self-defined needs. Promotion materials should be tailored to each specific population to minimize the potential for confusion about eligibility. Mixed methods longitudinal approaches.
Wood et al. (2022b) USA	Help-seeking and help-provision	Mixed design 352 service providers; race/ethnicity included	DV and sexual assault staff have more personal and professional stressors, are challenged by practice adaptations, perceive that their client safety has decreased, and lack the needed resources to help survivors.	Provide training, infrastructure, and support for virtual DV and sexual assault services. Address occupational stressors during the COVID-19 pandemic. Evaluate virtual services. Understand adaptations in safety planning during stay-at-home and social distancing. Examine the impact of work conditions on worker stress and anticipate post-pandemic shifts.

*Note.* DV = domestic violence; ER = emergency room; GBMSM = gay, bisexual, and other men who have sex with men; WHO = World Health Organization.

## Results

### Descriptive Characteristics

The descriptive characteristics of the eligible studies are presented in Table 1. Twenty-six studies presented data from the Americas region (North America: United States *n* = 20, Canada *n* = 2, and Mexico *n* = 1; South America: Peru *n* = 1 and Brazil *n* = 2), 15 from the Asian region (South Asia: India *n* = 4, Jordan *n* = 3, Nepal *n* = 1, Bangladesh *n* = 1, and Pakistan *n* = 1; West Asia: Lebanon *n* = 1, Nigeria *n* = 1, Iraq *n* = 1, and Iran *n* = 1; East Asia: Korea *n* = 1), 8 from Europe (Spain *n* = 2, United Kingdom *n* = 2, Italy *n* = 1, Sweden *n* = 1, Netherlands *n* = 1, and Germany *n* = 1), 5 from the African region (East Africa: Ethiopia *n* = 3; North Africa (Egypt *n* = 1 and Tunisia *n* = 1), and 3 from the Asia-Pacific region (Australia *n* = 2 and New Zealand *n* = 1). In an investigation of victims’ encounters with intimate partner violence during the lockdown and the COVID-19 pandemic, forum posts from the United States, the United Kingdom, Canada, Australia, and Cambodia were analyzed. It is important to note that, in the majority of cases, identifying the exact country of origin for these forum posts was not feasible (Lyons & Brewer, 2022).

The majority of studies (*n* = 47) undertaken to address their research objectives employed quantitative research designs (Abdel Rahman, 2021; Abuhammad, 2021; Abujilban et al., 2022; Agüero, 2021; Akel et al., 2022; Aolymat, 2021; Arenas-Arroyo et al., 2021; Cannon et al., 2021; E. S.Chang & Levy, 2021; Y. R.Chang et al., 2020; de la Miyar et al., 2021; Di Franco et al., 2021; Evans et al., 2021; Every-Palmer et al., 2020; García-Fernández et al., 2021; Gebrewahd et al., 2020; Gerell et al., 2020; Ghimire et al., 2020; Gosangi et al., 2021; Hamadani et al., 2020; Haq et al., 2020; Holland et al., 2021; Hsu & Henke, 2022; Jetelina et al., 2021; Jung et al., 2020; Krishnamurti et al., 2021; Leslie & Wilson, 2020; Mahmood et al., 2022; McLay, 2022; Mohler et al., 2020; Morgan & Boxall, 2020; Muldoon et al., 2021; Naghizadeh et al., 2021; Nix & Richards, 2021; Olding et al., 2021; Pal et al., 2021; Payne et al., 2020; Piquero et al., 2020; Raj et al., 2020; Rhodes et al., 2020; Ribeiro-Junior et al., 2021; Sediri et al., 2020; Sharma & Khokhar, 2022; Singh, 2020; Stephenson et al., 2022; Tadesse et al., 2022; Teshome et al., 2021). Eight studies utilized qualitative methodologies to delve into the nuanced aspects of the phenomena under investigation (Campos et al., 2020; Fawole et al., 2021; Koshan et al., 2020; Lyons & Brewer, 2022; Maji et al., 2022; Nnawulezi & Hacskaylo, 2022; Sabri et al., 2020; Speed et al., 2020). Three studies in this collection employed a mixed design approach, combining both qualitative and quantitative research methodologies (Tierolf et al., 2021; Wood et al., 2022a, 2022b).

Furthermore, a substantial portion of the studies (*n* = 27) employed a community-based sample (Abdel Rahman, 2021; Abuhammad, 2021; Abujilban et al., 2022; Akel et al., 2022; Aolymat, 2021; Arenas-Arroyo et al., 2021; Cannon et al., 2021; E. S.Chang & Levy, 2021; Every-Palmer et al., 2020; Gebrewahd et al., 2020; Ghimire et al., 2020; Hamadani et al., 2020; Haq et al., 2020; Jetelina et al., 2021; Jung et al., 2020; Krishnamurti et al., 2021; Mahmood et al., 2022; Morgan & Boxall, 2020; Naghizadeh et al., 2021; Pal et al., 2021; Raj et al., 2020; Sediri et al., 2020; Sharma & Khokhar, 2022; Singh, 2020; Stephenson et al., 2022; Tadesse et al., 2022; Teshome et al., 2021). These studies predominantly utilized survey instruments created by the researchers or employed standardized scales, reflecting a methodological emphasis on gathering data directly from community participants to capture their experiences and perspectives in the context of the COVID-19 pandemic. Numerous studies incorporated data from a variety of reports, offering diverse perspectives and information. These studies utilized a diverse array of data sources, including health reports (*n* = 9;Di Franco et al., 2021; Gosangi et al., 2021; Holland et al., 2021; Muldoon et al., 2021; Olding et al., 2021; Rhodes et al., 2020; Ribeiro-Junior et al., 2021; Y.R.Chang et al., 2020), police reports (*n* = 8; Evans et al., 2021; Gerell et al., 2020; Hsu & Henke (2022); Leslie & Wilson, 2020; McLay, 2022; Mohler et al., 2020; Nix & Richards, 2021; Piquero et al., 2020; Ribeiro-Junior et al., 2021), reports from help services (*n* = 2; Agüero, 2021; Fawole et al., 2021), court reports (*n* = 2; de la Miyar et al., 2021; Koshan et al., 2020), media reports (*n* = 2; Fawole et al., 2021; Maji et al., 2022), and governmental data (*n* = 1; Payne et al., 2020). Two studies, conducted by Fawole et al. (2021) and Ribeiro-Junior et al. (2021), employed distinct records as data sources. Fawole et al. (2021) utilized reports from both media and help services, while Ribeiro-Junior et al. (2021) incorporated reports from police and health sources in their analyses. Four studies employed victim-based samples, focusing on individuals who have experienced specific incidents or circumstances related to the topics under investigation (Lyons & Brewer, 2022; Sabri et al., 2020; Tierolf et al., 2021; Wood et al., 2022a). Finally, six studies employed samples derived from service providers (Campos et al., 2020; Nnawulezi & Hacskaylo, 2022; Sabri et al., 2020; Speed et al., 2020; Tierolf et al., 2021; Wood et al., 2022b). Two studies conducted by Sabri et al. (2020) and Tierolf et al. (2021) employed victim-based and service-provider samples.

### Geographic Analysis: Results by Regions

#### North America

##### Incidence of Domestic Violence

The occurrence of domestic violence in the United States during the COVID-19 pandemic exhibited diverse patterns across multiple investigations. Notably, prevalence rates varied between 10.4% and 18%, as highlighted by Cannon et al. (2021), Jetelina et al. (2021), Raj et al. (2020), and Stephenson et al. (2022), indicating a modest increase compared to prior reports (e.g., 12% as reported by Smith et al., 2018). Correspondingly, Holland et al. (2021) documented heightened domestic violence levels during the pandemic, while Hsu and Henke (2022) observed a 5% uptick in violence coinciding with stay-at-home orders. Leslie and Wilson (2020) reported a noteworthy 10.5% surge in service calls, particularly within the initial 5 weeks. Evans et al. (2021) noted an 11% escalation in domestic violence subsequent to shelter-in-place orders. Rhodes et al. (2020) documented an increase in assaults during lockdown, and Sabri et al. (2020) observed amplified frequency and severity of domestic violence. E. S.Chang and Levy (2021) found a 21.3% prevalence of domestic violence among individuals aged 60 years or older during the pandemic, an 83.6% increase from previous studies. Incidents were reported as occurring more frequently (24.9%) compared to before. Specific elder violence subtypes also increased during the pandemic, with a 114.3% increase in financial mistreatment and a 237.5% increase in physical violence; verbal abuse remained consistent ( E. S.Chang & Levy, 2021). Piquero et al. (2020) noted an initial upswing followed by a subsequent decrease in domestic violence following stay-at-home orders. Krishnamurti et al. (2021) identified comparable levels of violence before and during shelter-in-place, with a slight increase. Gosangi et al. (2021) documented a general decline in reported cases but underscored a troubling rise in physical incidents. Conversely, in Canada, a notable contrast emerged, revealing a substantial increase in psychological abuse compared to sexual and physical abuse, as reported by Muldoon et al. (2021). Also, McLay (2022) indicated a marginal decrease in cases during the pandemic; the same was seen in Mexico (de la Miyar et al., 2021), where a decrease in domestic violence reports was seen.

##### Risk Factors and Major Concerns

Exploring the multifaceted landscape of risk factors associated with domestic violence during the COVID-19 pandemic, researchers have identified various dimensions. Cannon et al. (2021) shed light on factors such as loss of income, renter status, and nutritional stress, emphasizing the economic and social complexities influencing vulnerability. Jetelina et al. (2021) pinpointed job/income changes as a significant risk factor, while McLay (2022) intriguingly noted a lower likelihood of cases with arrests. E. S.Chang and Levy (2021) found that younger older adults, larger households, poorer health, diminished sense of community, lower adherence to physical distancing, and higher levels of financial strain were risk factors for elder abuse during the COVID-19 pandemic. Mental health emerged as a key facet, with Raj et al. (2020) linking domestic violence to mental health symptoms. Sabri et al. (2020) highlighted the impact of partners being at home and increased stalking/control. Stephenson et al. (2022) delved into risk factors including substance use, skipping meals, and heightened anxiety. Wood et al. (2022a) broadened the perspective, outlining safety concerns related to violence and broader economic and health challenges such as unemployment, food insecurity, and childcare difficulties. This comprehensive understanding of risk factors and their broader implications contributes to a more nuanced approach to addressing domestic violence during challenging times.

##### Help-Seeking and Help-Provision

Concerns among service providers amid the pandemic were brought to light in the study conducted by Wood et al. (2022b) and Koshan et al. (2020). Wood et al. (2022b) detailed the challenges faced by domestic violence and sexual assault staff, encompassing both personal and professional obstacles. These challenges included perceived decreases in client safety, along with a notable shortage of essential resources for aiding survivors. In a related vein, Koshan et al. (2020) highlighted a pervasive lack of awareness regarding the heightened risks for survivors during the COVID-19 crisis, emphasizing the pressing need for increased awareness and support mechanisms.

#### South America

##### Incidence of Domestic Violence

While Agüero (2021) reported a substantial 48% increase in the incidence rate of calls between April and July 2020 in Peru, Ribeiro-Junior et al. (2021) observed a contrasting trend, noting a decrease in domestic violence during the first half of 2020 in Brazil. Interestingly, this reduction persisted even as COVID-19 cases continued to rise, highlighting the complexity of the impact of the pandemic on domestic violence dynamics.

##### Help-Seeking and Help-Provision

Amid the challenges posed by the COVID-19 pandemic, Campos et al. (2020) shed light on the heightened social vulnerability experienced by women. The study revealed that the pandemic intensified existing vulnerabilities, underscoring the pressing need for targeted support. In response to the evolving circumstances, protective services implemented new emergency measures and care protocols, demonstrating adaptability in their approach to address the unique challenges presented by the pandemic. However, an unfortunate consequence emerged in the form of increased deprivation of remote communication, potentially hindering access to crucial support services. This dual dynamic of heightened vulnerability and adaptive measures in protective services emphasizes the importance of ongoing efforts to enhance help-seeking avenues and ensure that support mechanisms remain accessible, particularly during times of crisis such as the COVID-19 pandemic.

#### Asia Region

##### Incidence of Domestic Violence

Studies settled in Asia reported higher values, having found a prevalence of 7.4% (India; Sharma & Khokhar, 2022), 18% (Nepal; Ghimire et al., 2020), 31% (Korea; Y. R.Chang et al., 2020), 35% (Pakistan and Iran; Haq et al., 2020; Naghizadeh et al., 2021), 38.7% (Iraq; Mahmood et al., 2022), 40% (Abuhammad, 2021), and 89.7% (Hamadani et al., 2020), which in the last three studies were almost two times and four times higher than previous studies (e.g., 18%; Smith et al., 2017). In India, Maji et al. (2022) and Pal et al. (2021) observed substantial increases in domestic violence cases during the pandemic and lockdown periods, respectively, indicating a concerning surge in violence. Singh (2020) highlighted the prevalence of high levels of domestic violence, encompassing physical harassment, ill-treatment, and abuse experienced by women in India. Moving to Jordan, Abujilban et al. (2022) noted higher incidences of violence before the quarantine, while Aolymat (2021) reported a significant 20.5% increase in domestic abuse during the pandemic. In Bangladesh, Hamadani et al. (2020) identified marked increases in emotional, physical, and sexual violence since the lockdown. Similarly, Haq et al. (2020) in Pakistan found that 17% of respondents faced emotional violence up to 12 times. Lebanon experienced substantial rates of abuse, with Akel et al. (2022) reporting 41.9% physical, 40.7% psychological, and 38.4% sexual abuse. Fawole et al. (2021) in Nigeria noted that while domestic violence was prevalent before the lockdown, its severity increased or involved new types of violence. In Iran, Naghizadeh et al. (2021) identified disturbing figures, with 32.8% experiencing emotional, 12.4% sexual, and 4.8% physical violence during the pandemic.

##### Risk Factors and Major Concerns

The intricate interplay between risk factors and domestic violence during the COVID-19 pandemic is evident across diverse countries in Asia. In India, Pal et al. (2021) discovered a significant association between job loss due to the lockdown and increased reports of domestic violence. Singh (2020) identified risk factors in India, including financial crisis, extramarital affairs, and dominant personality traits. Moving to Jordan, Abuhammad (2021) highlighted unemployment and marital issues as key predictors of domestic violence, while Abujilban et al. (2022) emphasized factors like marital conflict, verbal fighting, and a lack of understanding as contributing to the risk of violence. In Nepal, Ghimire et al. (2020) outlined risk factors such as lower age groups, unmarried individuals living with a spouse, lower education levels, and a history of mental disorders, with poor mental well-being and increased substance consumption during lockdown being associated with victimization. In Pakistan, Haq et al. (2020) identified a range of risk factors, including extended households, higher numbers of children, strained relationships, unemployment, anxiety, and disempowerment. Akel et al. (2022) in Lebanon found higher levels of anxiety, stress, depression, and low self-esteem contributing to domestic violence. Fawole et al. (2021) in Nigeria revealed that stressors such as custody disputes, disrupted income, and economic stress during the lockdown heightened the risk of violence, disrupting women’s support networks. In Iran, Naghizadeh et al. (2021) pinpointed self-rated health status, reduced spouse income, aggressive spouse behavior, and the impact of the disease on the relationship as risk factors for domestic violence, with a consequential decrease in the mental health dimension of the quality of life among those exposed to violence.

#### Europe

##### Incidence of Domestic Violence

The impact of the COVID-19 pandemic on domestic violence varied across European countries. In Spain, Arenas-Arroyo et al. (2021) reported a concerning 23% increase in violence during the lockdown, highlighting the escalation of domestic violence in this period. Moving to the United Kingdom, Olding et al. (2021) observed a rise in injuries resulting from domestic violence during the lockdown period, even though the proportion of domestic violence cases among total penetrating trauma decreased. Italy, as reported by Di Franco et al. (2021), had a notably low diagnosis rate of domestic violence, constituting only 0.6% of emergency admissions. In Sweden, Gerell et al. (2020) noted a drop in indoor violence during weeks 9–11 of the lockdown, stabilizing at a level slightly lower than expected thereafter. In the Netherlands, Tierolf et al. (2021) found no significant difference in violence before and after the lockdown, indicating a persistence of frequent or serious violence. Germany, as investigated by Jung et al. (2020), witnessed 5% of participants experiencing domestic violence during the pandemic, with women reporting more verbal, physical, and sexual violence compared to men.

##### Risk Factors and Major Concerns

The impact of the COVID-19 pandemic on domestic violence and associated factors reveals a complex interplay across European nations. In Spain, Arenas-Arroyo et al. (2021) identified risk factors for domestic violence during the lockdown, highlighting the association with economic stress and the confinement of at least one member of the couple. Meanwhile, García-Fernández et al. (2021) in Spain found that females who experienced domestic violence exhibited significantly higher levels of depressive symptoms compared to those who had not, underscoring the profound mental health consequences of domestic violence during the pandemic. In Italy, Di Franco et al. (2021) shed light on the multifaceted impact of the pandemic, with the highest percentages of adverse outcomes such as abortions, impairment of daily activities, psychosomatic symptoms, and anxious-depressive symptoms linked to domestic violence. In the Netherlands, Tierolf et al. (2021) noted the pervasive influence of the coronavirus crisis, which consistently altered the life situations of family members, often leading to heightened stress within families.

##### Help-Seeking and Help-Provision

The provision of support services for victims of domestic violence during the COVID-19 pandemic in the United Kingdom and the Netherlands faced notable challenges. Speed et al. (2020) in the United Kingdom reported both positive and concerning aspects of service capacity during the outbreak. While some organizations increased their capacity by extending service hours and providing telephone helplines or online facilities, a reduction or withdrawal of support services raised concerns about compromised justice for victims. Only 17 of 40 organizations continued to offer support, indicating a potential gap in assistance for those in need. Accessing justice became a significant worry, with potential delays in criminal trials and a lack of communication with victims. In the Netherlands, Tierolf et al. (2021) highlighted a shift in the format of help provision, with services adapting to the circumstances by employing video calls and telephone communication. These findings underscore the critical importance of maintaining and adapting support services during challenging times, ensuring continued accessibility and effectiveness for individuals experiencing domestic violence.

#### Africa Region

##### Incidence of Domestic Violence

The prevalence and impact of domestic violence during the COVID-19 pandemic in African nations shed light on concerning trends. In Ethiopia, Tadesse et al. (2022) reported an overall prevalence of at least one form of domestic violence at 22.4%, highlighting the widespread nature of this issue. Teshome et al. (2021) revealed that 9.5% of individuals in Ethiopia had experienced domestic violence within the year of the interview, with a notable 20.4% reporting an increase in violence after pregnancy and 18.2% perceiving heightened violence following the onset of the COVID-19 outbreak. Gebrewahd et al. (2020) similarly found a high prevalence of domestic violence against women in Ethiopia, reaching 24.6%. This is consistent with values found prior to the pandemic in Ethiopia (e.g., 19.2%–78.0% lifetime domestic violence against women; Semahegn & Mengistie, 2015). In Egypt, Abdel Rahman (2021) identified elevated levels of domestic violence during the pandemic, emphasizing the regional impact of the crisis on interpersonal relationships. Moving to Tunisia, Sediri et al. (2020) observed a significant increase in violence against women during the lockdown, surging from 4.4% to 14.8%.

##### Risk Factors and Major Concerns

The dynamics and predictors of domestic violence in the African context, particularly in Ethiopia and Egypt, present a complex interplay of sociodemographic factors and pre-existing conditions. In Ethiopia, Tadesse et al. (2022) identified several risk factors for domestic violence, including illiteracy, having an illiterate husband, substance use, and a community-tolerant attitude toward violence. Teshome et al. (2021) highlighted drug and alcohol use as predictors of domestic violence in Ethiopia, underscoring the role of substance abuse in contributing to such incidents. Gebrewahd et al. (2020) identified risk factors such as being an uneducated housewife, aged younger than 30 years, women with arranged marriages, and having a husband aged “between” 31 and 40 years. In Egypt, Abdel Rahman (2021) explored the impact of years of marriage and sex as predictors of domestic violence during the pandemic. The study in Tunisia by Sediri et al. (2020) revealed that pre-pandemic violence was a significant predictor of violence during the lockdown, with individuals having a history of domestic violence experiencing a much higher rate of violence during the pandemic. Moreover, the association between domestic violence and higher scores of depression, anxiety, and stress highlights the psychological toll of violence in this context.

#### Asia-Pacific Region

##### Incidence of Domestic Violence

In Australia, Payne et al. (2020) observed that the rate at which domestic violence orders were breached remained unchanged. Nevertheless, another study developed in this area (Morgan & Boxall, 2020) found that 2.9% of women reported experiencing physical or sexual violence by their partner for the first time during the pandemic and 67% of those who had experienced prior violence reported a repeat act of physical or sexual violence during the pandemic. In New Zealand, Every-Palmer et al. (2020) reported that 9.1% of participants directly experienced some form of family harm during the lockdown period, with an additional 3.9% witnessing family harm in which they were not the victim.

##### Risk Factors and Major Concerns

Morgan and Boxall (2020) identified risk factors and predictors in the Australian context, revealing that individuals with less frequent social interaction with friends or family, spending less time at home, and experiencing a higher level of financial stress were more susceptible to the occurrence of domestic violence during the pandemic.

#### Multinational Studies

Lyons and Brewer (2022) conducted a global analysis, identifying common themes among posts associated with domestic violence victims’ experiences during lockdown and the global pandemic. The identified themes included the abuser’s use of COVID-19 as a tool, such as making eviction threats, disruptions in essential services like shelters and counseling, delays in victims’ attempts to leave abusive situations, and various factors contributing to increased abuse or distress, such as financial stress and isolation. This comprehensive examination highlights the diverse challenges faced by domestic violence victims across different regions during the pandemic.

### Recommendations for Practice and Policy

The screening and assessment of domestic violence cases felled on frontline professionals (e.g., law enforcement, healthcare professionals, and social professionals), as well as in neighbors and communities during COVID-19 pandemic quarantines and shelter-in-place mandates. For example, several studies advocated for improved public awareness about the risk factors and warning signs of domestic violence (Akel et al., 2022; Cannon et al., 2021; E. S.Chang & Levy, 2021; Evans et al., 2021; Fawole et al., 2021; Holland et al., 2021; Jetelina et al., 2021; Lyons & Brewer, 2022; Mahmood et al., 2022; Rhodes et al., 2020; Sharma & Khokhar, 2022; Singh, 2020). Some authors cited the need for professionals to cooperate among themselves and with the public to improve domestic violence assessments and interventions during the COVID-19 pandemic (Campos et al., 2020; Cannon et al., 2021; Gebrewahd et al., 2020; Mahmood et al., 2022; Maji et al., 2022; Muldoon et al., 2021; Nix & Richards, 2021; Sediri et al., 2020).

The need for new national and institutional policies during the pandemic crises was also discussed. Those policies help reinforce and expand these cooperative strategies and develop new prevention, identification, and support approaches (Agüero, 2021; Akel et al., 2022; Evans et al., 2021; Every-Palmer et al., 2020; Haq et al., 2020; Maji et al., 2022; Naghizadeh et al., 2021). Approaches should include evidence-based measures and special resources/incentives to address violence in national response plans during and after the COVID-19 pandemic (Abujilban et al., 2022; Evans et al., 2021; Hamadani et al., 2020; Hsu & Henke, 2022; Jung et al., 2020; Koshan et al., 2020; Lyons & Brewer, 2022; Nnawulezi & Hacskaylo, 2022; Rhodes et al., 2020; Sabri et al., 2020; Sediri et al., 2020). Examples included improving communication and public health approaches (e.g., improving helplines responses and communication, public campaigns, enhancing gender equality, and community- and institution-based approaches; Abuhammad, 2021; Evans et al., 2021; Sabri et al., 2020; Sharma & Khokhar, 2022; Speed et al., 2020; Tadesse et al., 2022), providing housing and economic stability (Evans et al., 2021; Hamadani et al., 2020; Hsu & Henke (2022); Nnawulezi & Hacskaylo, 2022; Raj et al., 2020), providing individualized and flexible services in all areas (Koshan et al., 2020; Nnawulezi & Hacskaylo, 2022), supporting victims to recognize/respond to different forms/levels of violence and to ensure that victims are aware of exceptions to social distancing (Lyons & Brewer, 2022). Also, funding should be directed to service providers to support and help victims leaving violent situations (Jetelina et al., 2021; McLay, 2022; Naghizadeh et al., 2021), and toward (comprehensive and culturally) specific services (Sabri et al., 2020; Speed et al., 2020; Wood et al., 2022b). Additionally, strategies should include the empowerment and reinforcement of (non)governmental organizations and their inclusion in policy decisions (Akel et al., 2022; Cannon et al., 2021; Hamadani et al., 2020).

Since the healthcare system may have a privileged place in the identification and assessment of violence during the pandemic, the reproductive and health systems should take important and proactive measures to reduce the risk of violence in addition to other sectors that usually intervene within this population (e.g., mental health, social, and judicial support services; Aolymat, 2021; Di Franco et al., 2021; Fawole et al., 2021; Gosangi et al., 2021; Krishnamurti et al., 2021; Naghizadeh et al., 2021). Such strategies include the implementation of screening protocols (e.g., traumatic injuries, psychosomatic symptoms, and anxious-depressive symptoms; Di Franco et al., 2021; E. S.Chang & Levy, 2021; Sabri et al., 2020; Sharma & Khokhar, 2022), include the proactive participation of other care providers that usually do not intervene with victims (e.g., radiologists; Gosangi et al., 2021), and consider the unique needs of pregnant women mitigating pregnancy-related risks (Krishnamurti et al., 2021). The need to identify and target vulnerable groups (e.g., based on gender, sexual orientation, economic status, migrant situation, prior violence, and other sociodemographic information; Abujilban et al., 2022; Arenas-Arroyo et al., 2021; Every-Palmer et al., 2020; García-Fernández et al., 2021; Ghimire et al., 2020; Gosangi et al., 2021; McLay, 2022; Olding et al., 2021; Raj et al., 2020; Stephenson et al., 2021; Wood et al., 2022a) was also emphasized.

Some authors highlighted the need for (non)governmental agencies to implement new evidence-based response/intervention programming (Abdel Rahman, 2021; Holland et al., 2021; Krishnamurti et al., 2021; Raj et al., 2020; Rhodes et al., 2020; Sabri et al., 2020). Suggestions for practice included the development of psychological support services (Abdel Rahman, 2021; Every-Palmer et al., 2020; Sediri et al., 2020; Y. R.Chang et al., 2020) through traditional and e-therapies and telehealth support (Abdel Rahman, 2021; Every-Palmer et al., 2020; Holland et al., 2021; Krishnamurti et al., 2021; Raj et al., 2020) while ensuring that the technical conditions are in place (Tierolf et al., 2021). Agencies are encouraged to develop prevention and intervention educational programs directed to professionals in all areas and society in general (Abuhammad, 2021; Haq et al., 2020; Jetelina et al., 2021; Koshan et al., 2020; Lyons & Brewer, 2022; Maji et al., 2022; Nix & Richards, 2021; Sabri et al., 2020; Sediri et al., 2020). Authors indicated that workers should gain competence in domestic violence increased risk, trauma-informed care and interviewing techniques, and cultural intricacies among immigrant victims (Jetelina et al., 2021; Lyons & Brewer, 2022; Nix & Richards, 2021; Sharma & Khokhar, 2022). Organizations should also give training in digital skills while ensuring that professionals have access to guidelines and information about digital contact (e.g., about privacy or the extent to which assistance could be provided; Tierolf et al., 2021; Wood et al., 2022a, 2022b). Societies should be educated and prepared to shatter the existing power hierarchy based on gender (Maji et al., 2022). Given the potential negative consequences of responding to domestic violence incidents, a trauma-informed organizational culture should be implemented, and workers should have access to support services (e.g., mental health; Nnawulezi & Hacskaylo, 2022; Nix & Richards, 2021; Wood et al., 2022b).

### Recommendations for Research

The literature also highlighted key recommendations for future research that would advance this field of study. Few studies called for researchers to address the impact of the COVID-19 pandemic on the mental health of victims and on other areas related to domestic violence (e.g., related injuries, women’s empowerment, and mental health problems, evaluation of services, and impact on workers; Evans et al., 2021; Hamadani et al., 2020; Olding et al., 2021; Wood et al., 2022b; Y. R.Chang et al., 2020). The need for similar and comparative investigations in other countries was also discussed (Gerell et al., 2020; Haq et al., 2020), along with the importance of incorporating additional data and analysis to examine the impact of the COVID-19 pandemic on domestic violence incidents (e.g., hotline data, law enforcement data, emergency medical services, neighborhood-level changes, the role of social media in domestic violence communication ecologies, understand if financial aid help reduces domestic violence during the pandemic, account for seasonal and other variations; Agüero, 2021; Cannon et al., 2021; Every-Palmer et al., 2020; Holland et al., 2021; McLay, 2022; Mohler et al., 2020; Nix & Richards, 2021; Payne et al., 2020; Raj et al., 2020; Rhodes et al., 2020; Ribeiro-Junior et al., 2021). Authors cited the need to study the effectiveness of specific interventions and services in this population to guide practice (e.g., culture-sensitive interventions, online counseling, and digital services; Abdel Rahman, 2021; Cannon et al., 2021; Maji et al., 2022; Pal et al., 2021; Sabri et al., 2020).

The literature also called for researchers to address methodological problems (e.g., generalization; Mohler et al., 2020) by including larger, random, and more representative samples (Fawole et al., 2021; Ghimire et al., 2020; Jetelina et al., 2021) and by controlling social desirability effects (Jung et al., 2020). Some authors demonstrated the importance of including all social classes and priority/minority groups like children, adolescents, women, refugees, those in same-sex relationships, and men in relationships with female perpetrators from an intersectional framework point of view (Abujilban et al., 2022; Every-Palmer et al., 2020; Ghimire et al., 2020; Jetelina et al., 2021; Lyons & Brewer, 2022; Mahmood et al., 2022; Stephenson et al., 2021). Further recommendations included the need for different research methodologies (e.g., qualitative, quantitative, or mixed-methods design, Difference-in-Differences study designs; Abuhammad, 2021; Every-Palmer et al., 2020; Fawole et al., 2021; Hamadani et al., 2020; Jetelina et al., 2021; Muldoon et al., 2021; Payne et al., 2020; Tadesse et al., 2022), and longitudinal studies (Ghimire et al., 2020; Raj et al., 2020; Wood et al., 2022a). A final key recommendation is to increase open data sharing between research and practice (McLay, 2022).

## Discussion

The present integrative review systematically aimed to synthesize literature on the repercussions of the COVID-19 pandemic on domestic violence crime and to inform and enhance practice, policy-making, and future research. Overall, a consistent finding across global studies indicated an increase or high levels of domestic violence victimization since the onset of the pandemic. Various ecological factors were identified as potential explanations for this increase. At the individual level, lower physical and mental health was a risk factor for increased violence (Akel et al., 2022; E. S.Chang & Levy, 2021; García-Fernández et al., 2021; Naghizadeh et al., 2021; Raj et al., 2020). These physical and mental health challenges can reduce a person’s ability to cope with stress, leading to an increased likelihood of domestic violence. Additionally, the rise in substance use during the pandemic was another critical factor (Haq et al., 2020; Stephenson et al., 2021; Teshome et al., 2021). Substance abuse can impair judgment and increase aggression, making individuals more prone to perpetrating or becoming victims of domestic violence. Furthermore, lower education levels and illiteracy were associated with a higher risk of domestic violence (Gebrewahd et al., 2020; Tadesse et al., 2022). These factors can limit access to information and resources, reducing the ability to seek help or understand the risks and signs of domestic violence. At the relationship level, the pandemic’s lockdown measures forced families to spend more time together, often in confined spaces. Living in larger households, where increased stress and conflict can lead to domestic violence, was a risk factor identified (Abdel Rahman, 2021; Abujilban et al., 2022; E. S.Chang & Levy, 2021; Haq et al., 2020; Singh, 2020). Moreover, the lockdowns meant that victims had to spend more time with their abusers, increasing the risk of violence (Lyons & Brewer, 2022; Sabri et al., 2020). Finally, at the environmental level, financial stress was a well-documented risk factor for domestic violence (Cannon et al., 2021; Jetelina et al., 2021; Pal et al., 2021; Wood et al., 2022a), as it can have heightened tensions within households. Social distancing measures reduced the opportunities for social interaction, cutting off vital support networks for victims. Isolation can prevent victims from seeking help and support, making them more vulnerable to abuse (Morgan & Boxall, 2020; Tierolf et al., 2021). The pandemic also disrupted many support services, such as shelters, hotlines, and counseling services. This made it harder for victims to access the help they needed, exacerbating the impact of the pandemic on domestic violence (Campos et al., 2020; Fawole et al., 2021; Lyons & Brewer, 2022; Speed et al., 2020; Tierolf et al., 2021). In some communities, there is a cultural or social tolerance toward domestic violence. This can be exacerbated in times of crisis as community resources and attention may be diverted to other issues, reducing the focus on preventing and addressing domestic violence and enhancing the risk of violence (Gebrewahd et al., 2020; Tadesse et al., 2022).

These ecological risk factors for domestic violence can also serve as barriers to help-seeking, exacerbating the challenges victims face in reaching out for support and escaping abusive situations. These barriers may also help explain the disparate outcomes observed in other studies within the same regions (e.g., Europe). For example, at the individual level, lower mental health (e.g., depression, and low self-esteem; Akel et al., 2022; Raj et al., 2020) can affect a victim’s ability to seek help. At the relationship levels, imbalances of power within relationships can deter victims from reporting abuse due to fear of retaliation or loss of control (Sabri et al., 2020). Additionally, as most complaints are self-reported by victims, the reporting of domestic violence during lockdowns depends on victims’ ability to make complaints while sharing domestic spaces with perpetrators (Ratnam, 2020, cited in Kumar, 2020). At the environmental level, governmental restrictions due to COVID-19 compelled victims to reduce contact with individuals outside their domestic sphere, including formal and informal support networks (Sacco et al., 2020). The pandemic’s impact on face-to-face interactions hindered victims from accessing support and posed challenges for professionals in providing necessary responses to such cases (Cortis et al., 2021), reinforcing feelings of helplessness among victims. Indeed, while this review showed that service adaptations such as extended hours, virtual consultations, and new emergency measures were implemented to address the evolving needs (Speed et al., 2020; Tierolf et al., 2021), professionals faced challenges when supporting their clients (e.g., decreased client safety, resource shortages, and a lack of awareness about the heightened risks for survivors; Koshan et al., 2020; Wood et al., 2022b). Additionally, decreased police interventions and limited access to the justice system during quarantine hindered reporting and contributed to a lack of accountability (Telles et al., 2021). Essential forensic activities in domestic violence courts, such as forensic examinations of abuse victims, underwent significant changes (Esposito et al., 2022). Forensic practitioners used virtual platforms for evaluations to maintain health and safety and ensure the resolution of criminal and other forensic cases (Mulay et al., 2021). While research on the use of virtual evaluations is limited, findings suggest that the benefits outweigh the concerns (Mulay et al., 2021). These approaches can save resources for organizations and individuals, prevent delays in proceedings, and protect victims from exposure to their perpetrators (Mulay et al., 2021).

## Recommendations for Future Research

Considering that the effects of the COVID-19 pandemic on families are expected to extend beyond the “stay-at-home” orders, and the risks of family violence are likely to persist for some time (Humphreys et al., 2020), additional research is needed and recommended. A literature review revealed a dearth of mental health interventions tailored to domestic violence victims in the context of COVID-19 (Su et al., 2021). Consequently, intervention research emerges as a priority to determine effective strategies for addressing domestic violence amid future health crises. Moreover, exploring how victims of domestic violence perceive the measures adopted by support institutions during the COVID-19 pandemic is equally critical. This research is pivotal for ensuring the success of response strategies in combating domestic violence not only in the current crisis but also in future health emergencies. Understanding the perspectives of victims provides valuable insights that can inform the development and adaptation of support systems to better meet the needs of those experiencing domestic violence during challenging times.

Furthermore, acknowledging that comprehending the perspectives and experiences of perpetrators is essential for a comprehensive understanding of the impact of the COVID-19 pandemic on the phenomenon of domestic violence is a proactive approach. To fully address this complex issue, researchers should consider incorporating the viewpoints of perpetrators into their investigations during this period. This approach is particularly relevant as background risk factors, such as childhood history variables, sociodemographic characteristics, abuse, and family of origin problems, may pose challenges to investigation in adulthood (Costa et al., 2015). Examining perpetrators during the pandemic becomes crucial, given previous research indicating that changes in situational circumstances can influence perpetration levels. Factors such as loss of support, food security, housing, and substance use have been identified as impacting perpetration (Adhia et al., 2020), and these dynamics need to be considered in the context of the COVID-19 pandemic. Additionally, the heightened use of technologies during this period (Vargo et al., 2021) has provided perpetrators with new avenues, and research must recognize these technological dynamics within the context of abusive relationships. Gaining insights into the behaviors of perpetrators, considering the unique circumstances brought about by the pandemic, is vital for addressing and ultimately combating domestic violence in a post-pandemic era. By delving into the experiences and perspectives of both victims and perpetrators, researchers can contribute to a more nuanced and comprehensive understanding of the intricate dynamics at play in domestic violence during and after the COVID-19 pandemic.

Our review underscores a gap in studies addressing how the intersectionality of vulnerabilities may influence domestic violence during the COVID-19 pandemic. Despite emerging evidence indicating that members of minority groups often experience higher levels of domestic violence (as observed in studies by Stiles-Shields & Carroll, 2015 and Taft et al., 2009), these groups are underrepresented in the articles included in our review. Notably, immigrants face additional challenges, such as living illegally in the host country, lack of social support, social exclusion, and poverty, as highlighted by Gonçalves and Matos (2016). Similarly, race/ethnic minorities encounter issues like a shortage of resources, racism, discrimination, mistreatment, and cultural barriers, as identified by Taft et al. (2009). Gender/sexual minorities also confront unique challenges, including seeking shelter, discrimination, and mutual battering, as explored in the work of Stiles-Shields and Carroll (2015).

To address these gaps, future studies should explicitly adopt an intersectionality framework, as advocated by scholars like Nixon and Humphreys (2010), when analyzing the impact of the COVID-19 pandemic on domestic violence. This approach is crucial for understanding how support services can better cater to the needs of these diverse populations facing compounded vulnerabilities. Additionally, our review highlights a deficiency in domestic violence research among men in heterosexual relationships, despite indications that men may also be victims of domestic violence. Studies, such as those cited by Scott-Storey et al. (2022), underscore the importance of addressing limitations in interventions and expanding research efforts to encompass the experiences of men in heterosexual relationships. Closing this gap is essential for a more comprehensive understanding of domestic violence dynamics and ensuring that support services are inclusive and effective for all individuals, regardless of gender or sexual orientation.

Finally, recognizing the inherent stress associated with working with domestic violence victims is crucial, as highlighted by Maslach and Leiter (2016). The unique demands of this profession, coupled with the escalating workload during the pandemic, place professionals dealing with domestic violence at risk of developing various mental health problems. These issues include but are not limited to secondary traumatic stress, increased stress levels, sleep problems, and headaches, as noted by Joshi and Sharma (2020) and McLean and McIntosh (2021). Further research focusing on professionals working with domestic violence victims is imperative. This research should delve into the comprehensive impact on their mental health, quality of life, and working conditions. The scope of this investigation should extend across diverse sectors, including social services, healthcare, justice, and shelter organizations. Such research is essential for gaining a nuanced understanding of the challenges faced by professionals in different contexts and developing targeted support mechanisms to address their specific needs.

### Study Limitations

This study faced some limitations. This review only included empirical studies published in English, Portuguese, French, or Spanish that appeared in peer-reviewed journals. Therefore, this review may leave out several other studies (un)published about the impact of COVID-19 on the domestic violence phenomenon. In addition, while our search was comprehensive, it was not exhaustive—some outcomes could have been missed, especially the ones outside the scope of our review.

## Conclusions

The COVID-19 pandemic was a time of unique vulnerability yet an opportunity to address and investigate domestic violence using an ecological framework approach. Expanding the research on domestic violence will allow researchers to understand unique factors that may impact specific groups, which could have further implications for domestic violence prevention and designing specific interventions for those groups. Furthermore, institutions require robust ways to assess the success of practices, including impacts on staff and users of service, and existing studies do not generally cover the full picture of professionals’ and victims’ experiences with support services during this period. Indeed, since the impact that the pandemic had on domestic violence will long outlive the health crisis, existing research may be insufficient to clearly inform professionals/institutional practices and guide policy decisions regarding the support of domestic violence victims during and after the COVID-19 pandemic. Recognizing the mutual benefit of research-practice partnerships is vital to addressing and preventing domestic violence. Creating those engaging collaborative and participatory alliances may have long-lasting benefits for the health and safety of individuals, families, and communities. Tables 2 and 3 summarize the key findings and recommendations for practice, policy, and research.

**Table 2.** Summary of Critical Findings.

Critical Findings
• Overall, studies consistently reported an increase in domestic violence victimization. • The COVID-19 pandemic seems to have exacerbated pre-existing conditions of vulnerability. • The coronavirus crisis raised safety concerns related to violence and concerns with service access. • The increase in domestic violence during the pandemic is linked to ecological factors such as lower physical and mental health, rising substance use, and financial stress, which heightened individuals’ vulnerability. • Lockdowns exacerbated these issues by increasing confinement in homes, disrupting support services, and limiting victims’ access to help. • Studies highlighted barriers to help-seeking and increased workload during the pandemic associated with more personal and professional stressors.

**Table 3.** Summary of Implications for Practice, Policy, and Research.

Implications
• Improving public awareness about domestic violence. • Improving professional cooperation among themselves and with the public. • Developing further national and institutional policies during and after the pandemic crises. • Involving other sectors that usually do not intervene directly with this population. • Identifying and targeting vulnerable groups. • More educational programs for professionals and the public. • Promoting professional access to mental health services. • Addressing the impact of the COVID-19 pandemic on the mental health of victims and in other areas. • Developing similar and comparative investigations in other countries. • Incorporating additional data and analysis. • Studying the effectiveness of specific interventions and services. • Recognizing that the perspectives and experiences of perpetrators are required to fully address this problem. • Addressing methodological problems by including larger, random, and more representative samples. • Controlling social desirability effects. • Including all social classes and priority/minority groups. • Adopting different research methodologies (e.g., qualitative, quantitative, or mixed-methods design, Difference-in-Differences study designs). • Increasing open data sharing between research and practice.

## Supplemental Material

sj-docx-1-tva-10.1177_15248380241277788 – Supplemental material for Understanding the Dynamics of Domestic Violence During the First Year of the Pandemic: An Integrative Review Supplemental material, sj-docx-1-tva-10.1177_15248380241277788 for Understanding the Dynamics of Domestic Violence During the First Year of the Pandemic: An Integrative Review by Ana Cunha, Mariana Gonçalves and Marlene Matos in Trauma, Violence, & Abuse
