# Supplementary material for: Understanding the Dynamics of Domestic Violence During the First Year of the Pandemic: An Integrative Review
Source: Trauma Violence Abuse. 2024 Sep 24;26(1):118–37. doi: 10.1177/15248380241277788 (PMC11558936; doi:10.1177/15248380241277788)
Supplement: sj-docx-1-tva-10.1177_15248380241277788 – Supplemental material for Understanding the Dynamics of Domestic Violence During the First Year of the Pandemic: An Integrative Review [file sj-docx-1-tva-10.1177_15248380241277788.docx]

**Appendix A - Understanding the Dynamics of Domestic Violence During the First Year of the Pandemic: An Integrative Review**

Identified articles:

Abdel Rahman, D. E. (2021). Predictors of family violence through the COVID-19 pandemic: Structural equation modeling. *Journal of Public Affairs*, 1–10. <https://doi.org/10.1002/pa.2626>

Abuhammad, S. (2021). Violence against Jordanian Women during COVID-19 Outbreak. *International Journal of Clinical Practice*, *75*(3), 1–7. <https://doi.org/10.1111/ijcp.13824>

Abujilban, S., Mrayan, L., Hamaideh, S., Obeisat, S., & Damra, J. (2021). Intimate Partner Violence Against Pregnant Jordanian Women at the Time of COVID-19 Pandemic’s Quarantine. *Journal of Interpersonal Violence*. <https://doi.org/10.1177/0886260520984259>

Agüero, J. M. (2021). COVID-19 and the rise of intimate partner violence. *World Development*, *137*. <https://doi.org/10.1016/j.worlddev.2020.105217>

Akel, M., Berro, J., Rahme, C., Haddad, C., Obeid, S., & Hallit, S. (2021). Violence Against Women During COVID-19 Pandemic. *Journal of Interpersonal Violence*. <https://doi.org/10.1177/0886260521997953>

Aolymat, I. (2021). A cross-sectional study of the impact of COVID-19 on domestic violence, menstruation, genital tract health, and contraception use among women in Jordan. *American Journal of Tropical Medicine and Hygiene*, *104*(2), 519–525. <https://doi.org/10.4269/ajtmh.20-1269>

Arenas-Arroyo, E., Fernandez-Kranz, D., & Nollenberger, N. (2021). Intimate partner violence under forced cohabitation and economic stress: Evidence from the COVID-19 pandemic. *Journal of Public Economics*, *194*, 104350. <https://doi.org/https://doi.org/10.1016/j.jpubeco.2020.104350>

Campos, B., Tchalekian, B., & Paiva, V. (2020). Violence against women: Programmatic vulnerability in times of SARS-COV-2 / COVID-19 in Sao Paulo. *Psicologia e Sociedade*, *32*. <https://doi.org/10.1590/1807-0310/2020V32240336>

Cannon, C. E. B., Ferreira, R., Buttell, F., & First, J. (2021). COVID-19, Intimate Partner Violence, and Communication Ecologies. *American Behavioral Scientist*, *65*(7), 992–1013. <https://doi.org/10.1177/0002764221992826>

Chang, E. S., & Levy, B. R. (2021). High Prevalence of Elder Abuse During the COVID-19 Pandemic: Risk and Resilience Factors. *The American Journal of Geriatric Psychiatry*. <https://doi.org/https://doi.org/10.1016/j.jagp.2021.01.007>

Chang, Y. R., Kim, K. M., Kim, H. J., Kim, D. H., Kim, J., Noh, D., Ma, D. S., Yun, J., Yun, J. H., Lee, S. W., Choi, S. H., Heo, Y., & Chang, S. W. (2020). Osong Public Health and Impacts of social distancing during the covid-19 outbreaks in Korea: Level 1 trauma center data of domestic incidents and intentional injury. *Osong Public Health and Research Perspectives*, *11*(6), 345–350.

de la Miyar, J. R. B., Hoehn-Velasco, L., & Silverio-Murillo, A. (2021). Druglords don’t stay at home: COVID-19 pandemic and crime patterns in Mexico City. *Journal of Criminal Justice*, *72*. <https://doi.org/10.1016/j.jcrimjus.2020.101745>

Di Franco, M., Martines, G. F., Carpinteri, G., Trovato, G., & Catalano, D. (2020). Domestic violence detection amid the COVID-19 pandemic: the value of the WHO questionnaire in Emergency Medicine. *QJM : Monthly Journal of the Association of Physicians*. <https://doi.org/10.1093/qjmed/hcaa333>

Evans, D. P., Hawk, S. R., & Ripkey, C. E. (2021). Domestic Violence in Atlanta, Georgia Before and During COVID-19. *Violence and Gender*, *8*(3), 140–147. <https://doi.org/10.1089/vio.2020.0061>

Every-Palmer, S., Jenkins, M., Gendall, P., Hoek, J., Beaglehole, B., Bell, C., Williman, J., Rapsey, C., & Stanley, J. (2020). Psychological distress, anxiety, family violence, suicidality, and wellbeing in New Zealand during the COVID-19 lockdown: A cross-sectional study. *PLoS One*, *15*(11). <https://doi.org/10.1371/journal.pone.0241658>

Fawole, O. I., Okedare, O. O., & Reed, E. (2021). Home was not a safe haven: women’s experiences of intimate partner violence during the COVID-19 lockdown in Nigeria. *BMC Women’s Health*, *21*(1). <https://doi.org/10.1186/s12905-021-01177-9>

García-Fernández, L., Romero-Ferreiro, V., Padilla, S., David López-Roldán, P., Monzó-García, M., & Rodriguez-Jimenez, R. (2021). Gender differences in emotional response to the COVID-19 outbreak in Spain. *Brain and Behavior*, *11*(1). <https://doi.org/10.1002/brb3.1934>

Gebrewahd, G. T., Gebremeskel, G. G., & Tadesse, D. B. (2020). Intimate partner violence against reproductive age women during COVID-19 pandemic in northern Ethiopia 2020: a community-based cross-sectional study. *Reproductive Health*, *17*(1), 152. <https://doi.org/10.1186/s12978-020-01002-w>

Gerell, M., Kardell, J., & Kindgren, J. (2020). Minor covid-19 association with crime in Sweden. *Crime Science*, *9*(1), 1–9. <https://doi.org/10.1186/s40163-020-00128-3>

Ghimire, C., Acharya, S., Shrestha, C., Prabhat, K. C., Singh, S., & Sharma, P. (2020). Interpersonal violence during the COVID-19 lockdown period in Nepal: A descriptive cross-sectional study. *Journal of the Nepal Medical Association*, *58*(230), 751–757. <https://doi.org/10.31729/jnma.5499>

Gosangi, B., Park, H., Thomas, R., Gujrathi, R., Camden, P., Harris, M. B., Boland, G. W., & Rexrode, K. (2021). Exacerbation of Physical Intimate Partner Violence during COVID-19 pandemic. *Radiology*, *298*(1), E38–E45. <https://doi.org/https://doi.org/10.1148/radiol.2020202866>

Hamadani, J. D., Hasan, M. I., Baldi, A. J., Hossain, S. J., Shiraji, S., Bhuiyan, M. S. A., Mehrin, S. F., Fisher, J., Tofail, F., Tipu, S. M. M. U., Braat, S., & Pasricha, S. R. (2020). Immediate impact of stay-at-home orders to control COVID-19 transmission on socioeconomic conditions, food insecurity, mental health, and intimate partner violence in Bangladeshi women and their families: an interrupted time series. *The Lancet Global Health*, *8*(11), e1380–e1389. <https://doi.org/10.1016/S2214-109X(20)30366-1>

Haq, W., Raza, S. H., & Mahmood, T. (2020). The pandemic paradox: Domestic violence and happiness of women. *PeerJ*, *8*. <https://doi.org/10.7717/peerj.10472>

Holland, K. M., Jones, C., Vivolo-Kantor, A. M., Idaikkadar, N., Zwald, M., Hoots, B., Yard, E., D’Inverno, A., Swedo, E., Chen, M. S., Petrosky, E., Board, A., Martinez, P., Stone, D. M., Law, R., Coletta, M. A., Adjemian, J., Thomas, C., Puddy, R. W., Peacock, G, Dowling, N. F., & Houry, D. (2021). Trends in US Emergency Department Visits for Mental Health, Overdose, and Violence Outcomes Before and During the COVID-19 Pandemic. *JAMA Psychiatry*, *78*(4), 372–379. <https://doi.org/10.1001/jamapsychiatry.2020.4402>

Hsu, L. C., & Henke, A. (2020). COVID-19, staying at home, and domestic violence. *Review of Economics of the Household*, 1–11. <https://pubmed.ncbi.nlm.nih.gov/33235551/>

Jetelina, K. K., Knell, G., & Molsberry, R. J. (2021). Changes in intimate partner violence during the early stages of the COVID-19 pandemic in the USA. *Injury Prevention*, *27*(1), 93–97. <https://doi.org/10.1136/injuryprev-2020-043831>

Jung, S., Kneer, J., & Krüger, T. H. C. (2020). Mental Health, Sense of Coherence, and Interpersonal Violence during the COVID-19 Pandemic Lockdown in Germany. *Journal of Clinical Medicine*, *9*(11). <https://doi.org/10.3390/jcm9113708>

Koshan, J., Mosher, J., & Wiegers, W. (2020). COVID-19, the shadow pandemic, and access to justice for survivors of domestic violence. *Osgoode Hall LJ,* *57*(3), 739–800. <http://dx.doi.org/10.2139/ssrn.3698160>

Krishnamurti, T., Davis, A. L., Quinn, B., Castillo, A. F., Martin, K. L., & Simhan, H. N. (2021). Mobile remote monitoring of intimate partner violence among pregnant patients during the COVID-19 shelter-in-place Order: Quality improvement pilot study. *Journal of Medical Internet Research*, *23*(2). <https://doi.org/10.2196/22790>

Leslie, E., & Wilson, R. (2020). Sheltering in place and domestic violence: Evidence from calls for service during COVID-19. *Journal of Public Economics*, *189*, 104241. <https://doi.org/10.1016/j.jpubeco.2020.104241>

Lyons, M., & Brewer, G. (2021). Experiences of Intimate Partner Violence during Lockdown and the COVID-19 Pandemic. *Journal of Family Violence*, 1–9. <https://doi.org/10.1007/s10896-021-00260-x>

Mahmood, K. I., Shabu, S. A., M-Amen, K. M., Hussain, S. S., Kako, D. A., Hinchliff, S., & Shabila, N. P. (2021). The Impact of COVID-19 Related Lockdown on the Prevalence of Spousal Violence Against Women in Kurdistan Region of Iraq. *Journal of Interpersonal Violence*, 886260521997929. <https://doi.org/10.1177/0886260521997929>

Maji, S., Bansod, S., & Singh, T. (2021). Domestic violence during COVID-19 pandemic: The case for Indian women. *Journal of Community and Applied Social Psychology*. <https://doi.org/10.1002/casp.2501>

McLay, M. M. (2021). When “Shelter-in-Place” Isn’t Shelter That’s Safe: a Rapid Analysis of Domestic Violence Case Differences during the COVID-19 Pandemic and Stay-at-Home Orders. *Journal of Family Violence*, 1–10. <https://doi.org/10.1007/s10896-020-00225-6>

Mohler, G., Bertozzi, A. L., Carter, J., Short, M. B., Sledge, D., Tita, G. E., Uchida, C. D., & Brantingham, P. J. (2020). Impact of social distancing during COVID-19 pandemic on crime in Los Angeles and Indianapolis. *Journal of Criminal Justice*, *68*. <https://doi.org/10.1016/j.jcrimjus.2020.101692>

Morgan, A., & Boxall, H. (2020). Social isolation, time spent at home, financial stress and domestic violence during the COVID-19 pandemic. *Trends and Issues in Crime and Criminal Justice*, *609*. <https://doi.org/10.52922/ti04855>

Muldoon, K. A., Denize, K. M., Talarico, R., Fell, D. B., Sobiesiak, A., Heimerl, M., & Sampsel, K. (2021). COVID-19 pandemic and violence: rising risks and decreasing urgent care-seeking for sexual assault and domestic violence survivors. *BMC Medicine*, *19*(1). <https://doi.org/10.1186/s12916-020-01897-z>

Naghizadeh, S., Mirghafourvand, M., & Mohammadirad, R. (2021). Domestic violence and its relationship with quality of life in pregnant women during the outbreak of COVID-19 disease. *BMC Pregnancy and Childbirth*, *21*(1). <https://doi.org/10.1186/s12884-021-03579-x>

Nix, J., & Richards, T. N. (2021). The immediate and long-term effects of COVID-19 stay-at-home orders on domestic violence calls for service across six U.S. jurisdictions. *Police Practice and Research*. <https://doi.org/10.1080/15614263.2021.1883018>

Nnawulezi, N., & Hacskaylo, M. (2021). Identifying and Responding to the Complex Needs of Domestic Violence Housing Practitioners at the Onset of the COVID-19 Pandemic. *Journal of Family Violence*, 1–11. <https://doi.org/10.1007/s10896-020-00231-8>

Olding, J., Zisman, S., Olding, C., & Fan, K. (2021). Penetrating trauma during a global pandemic: Changing patterns in interpersonal violence, self-harm and domestic violence in the Covid-19 outbreak. *Surgeon*, *19*(1), e9–e13. <https://doi.org/10.1016/j.surge.2020.07.004>

Pal, A., Gondwal, R., Paul, S., Bohra, R., Aulakh, A. P. S., & Bhat, A. (2021). Effect of COVID-19–Related Lockdown on Intimate Partner Violence in India: An Online Survey-Based Study. *Violence and Gender*, *8*(3), 157–162. <https://doi.org/10.1089/vio.2020.0050>

Payne, J. L., Morgan, A., & Piquero, A. R. (2020). COVID-19 and social distancing measures in Queensland, Australia, are associated with short-term decreases in recorded violent crime. *Journal of Experimental Criminology*. <https://doi.org/10.1007/s11292-020-09441-y>

Piquero, A. R., Riddell, J. R., Bishopp, S. A., Narvey, C., Reid, J. A., & Piquero, N. L. (2020). Staying Home, Staying Safe? A Short-Term Analysis of COVID-19 on Dallas Domestic Violence. *American Journal of Criminal Justice : AJCJ*, 1–35. <https://doi.org/10.1007/s12103-020-09531-7>

Raj, A., Johns, N. E., Barker, K. M., & Silverman, J. G. (2020). Time from COVID-19 shutdown, gender-based violence exposure, and mental health outcomes among a state representative sample of California residents. *EClinicalMedicine*, *26*, 100520. <https://doi.org/10.1016/j.eclinm.2020.100520>

Rhodes, H. X., Petersen, K., Lunsford, L., & Biswas, S. (2020). COVID-19 Resilience for Survival: Occurrence of Domestic Violence During Lockdown at a Rural American College of Surgeons Verified Level One Trauma Center. *Cureus*, *12*(8), e10059. <https://doi.org/10.7759/cureus.10059>

Ribeiro-Junior, M. A. F., NÉder, P. R., Augusto, S. D. E. S., Elias, Y. G. B., Hluchan, K., & Santo-Rosa, O. M. (2021). Current state of trauma and violence in São Paulo - Brazil during the COVID-19 pandemic. *Revista Do Colegio Brasileiro de Cirurgioes*, *48*, e20202875. <https://doi.org/10.1590/0100-6991e-20202875>

Sabri, B., Hartley, M., Saha, J., Murray, S., Glass, N., & Campbell, J. C. (2020). Effect of COVID-19 pandemic on women’s health and safety: A study of immigrant survivors of intimate partner violence. *Health Care for Women International*, *41*(11–12), 1294–1312. <https://doi.org/10.1080/07399332.2020.1833012>

Sediri, S., Zgueb, Y., Ouanes, S., Ouali, U., Bourgou, S., Jomli, R., & Nacef, F. (2020). Women’s mental health: acute impact of COVID-19 pandemic on domestic violence. *Archives of Women’s Mental Health*, *23*(6), 749–756. <https://doi.org/10.1007/s00737-020-01082-4>

Sharma, P., & Khokhar, A. (2021). Domestic violence and coping strategies among married adults during lockdown due to Coronavirus disease (COVID-19) pandemic in India: a cross-sectional study. *Disaster Medicine and Public Health Preparedness*, 1–29. <https://doi.org/10.1017/dmp.2021.59>

Singh, M. (2020). Domestic harassment of women and children during COVID-19. *European Journal of Molecular and Clinical Medicine*, *7*(6), 754–761. <https://www.scopus.com/inward/record.uri?eid=2-s2.0-85096986618&partnerID=40&md5=75917cbab5adc54bf56e413ae6904640>

Speed, A., Thomson, C., & Richardson, K. (2020). Stay Home, Stay Safe, Save Lives? An Analysis of the Impact of COVID-19 on the Ability of Victims of Gender-based Violence to Access Justice. *The Journal of Criminal Law*, *84*(6), 539–572. <https://doi.org/10.1177/0022018320948280>

Stephenson, R., Chavanduka, T. M. D., Rosso, M. T., Sullivan, S. P., Pitter, R. A., Hunter, A. S., & Rogers, E. (2021). COVID-19 and the Risk for Increased Intimate Partner Violence Among Gay, Bisexual and Other Men Who Have Sex With Men in the United States. *Journal of Interpersonal Violence*, *May 2020*, 1–16. <https://doi.org/10.1177/0886260521997454>

Tadesse, A. W., Tarekegn, S. M., Wagaw, G. B., Muluneh, M. D., & Kassa, A. M. (2020). Prevalence and Associated Factors of Intimate Partner Violence Among Married Women During COVID-19 Pandemic Restrictions: A Community-Based Study. *Journal of Interpersonal Violence*. <https://doi.org/10.1177/0886260520976222>

Teshome, A., Gudu, W., Bekele, D., Asfaw, M., Enyew, R., & Compton, S. D. (2021). Intimate partner violence among prenatal care attendees amidst the COVID-19 crisis: The incidence in Ethiopia. *International Journal of Gynaecology and Obstetrics: The Official Organ of the International Federation of Gynaecology and Obstetrics*, *153*(1), 45–50. <https://doi.org/10.1002/ijgo.13566>

Tierolf, B., Geurts, E., & Steketee, M. (2021). Domestic violence in families in the Netherlands during the coronavirus crisis: A mixed method study. *Child Abuse and Neglect*, *116*(P2), 104800. <https://doi.org/10.1016/j.chiabu.2020.104800>

Wood, L., Baumler, E., Schrag, R. V., Guillot-Wright, S., Hairston, D., Temple, J., & Torres, E. (2021). “Don’t Know where to Go for Help”: Safety and Economic Needs among Violence Survivors during the COVID-19 Pandemic. *Journal of Family Violence*. <https://doi.org/10.1007/s10896-020-00240-7>

Wood, L., Schrag, R. V., Baumler, E., Hairston, D., Guillot-Wright, S., Torres, E., & Temple, J. R. (2020). On the Front Lines of the COVID-19 Pandemic: Occupational Experiences of the Intimate Partner Violence and Sexual Assault Workforce. *Journal of Interpersonal Violence*. <https://doi.org/10.1177/0886260520983304>
